# ABioTrans: A Biostatistical Tool for Transcriptomics Analysis

**DOI:** 10.3389/fgene.2019.00499

**Published:** 2019-05-31

**Authors:** Yutong Zou, Thuy Tien Bui, Kumar Selvarajoo

**Affiliations:** ^1^Department of Statistics and Applied Probability, National University of Singapore, Singapore, Singapore; ^2^Biotransformation Innovation Platform (BioTrans), Agency for Science, Technology and Research (A^*^STAR), Singapore, Singapore

**Keywords:** transcriptomics, correlation, entropy, noise, DEG (differentially expressed genes), RNA-seq, clustering, gene expression data

## Abstract

Here we report a bio-statistical/informatics tool, ABioTrans, developed in R for gene expression analysis. The tool allows the user to directly read RNA-Seq data files deposited in the Gene Expression Omnibus or GEO database. Operated using any web browser application, ABioTrans provides easy options for multiple statistical distribution fitting, Pearson and Spearman rank correlations, PCA, *k*-means and hierarchical clustering, differential expression (DE) analysis, Shannon entropy and noise (square of coefficient of variation) analyses, as well as Gene ontology classifications.

## Introduction

Large-scale gene expression analysis requires specialized statistical or bioinformatics tools to rigorously interpret the complex multi-dimensional data, especially when comparing between genotypes. There are already several such tools developed with fairly user-friendly features (Russo and Angelini, 2014; Poplawski et al., 2016; Velmeshev et al., 2016). Nevertheless, there still is a need for more specialized, focused and “click-and-go” analysis tools for different groups of bioinformatics and wet biologists. In particular, software tools that perform gene expression variability through entropy and noise analyses are lacking. Here, we focused on very commonly used statistical techniques, namely, Pearson and Spearman rank correlations, Principal Component Analysis (PCA), *k*-means and hierarchical clustering, Shannon entropy, noise (square of coefficient of variation), differential expression (DE) analysis, and gene ontology classifications (Tsuchiya et al., 2009; Piras et al., 2014; Piras and Selvarajoo, 2015; Simeoni et al., 2015).

Using R programming as the backbone, we developed a web-browser based user interface to simply perform the above-mentioned analyses by a click of a few buttons, rather than using a command line execution. Our interface is specifically made simple considering wet lab biologists as the main users. Nevertheless, our tool will also benefit bioinformatics and computational biologists at large, as it saves much time for running the R script files for analyses and saving the results in pdf.

## Main Interface and Data Input

**FIGURE 1:**
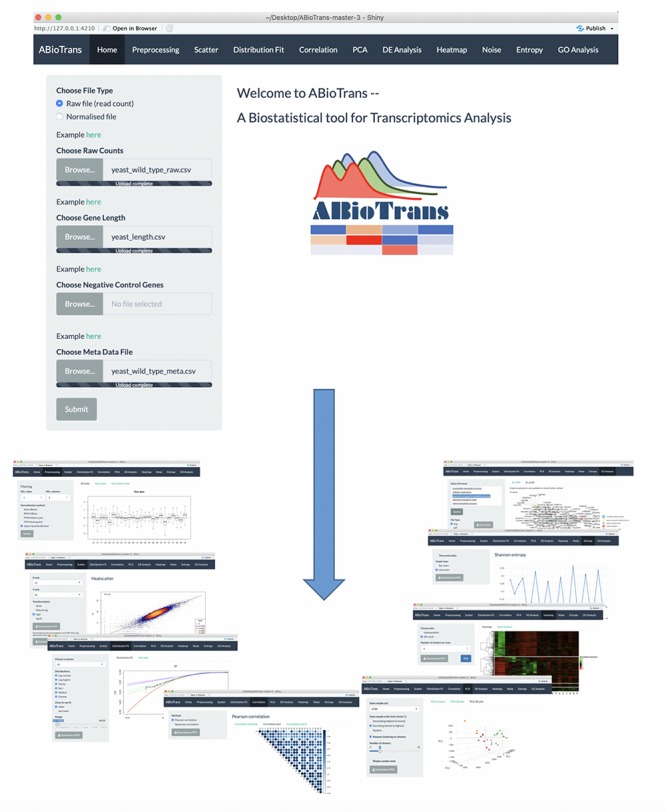
ABioTrans main interface and snapshots of various analysis mode.

Upon loading ABioTrans.R, the homepage window pops up and displays a panel to choose the RNA-Seq data and supporting files (Figure 1). The data file, in comma-separated value (.csv) format, should contain the gene names in rows and genotypes (conditions: wildtype, mutants, and replicates, etc.) in columns, following the usual format of files deposited in the GEO database (Clough and Barrett, 2016). Supporting files (if applicable) include gene length, list of negative

control genes, and metadata file. If the data files contain raw read counts, the user can perform normalization using 5 popular methods: FPKM, RPKM, TPM, Remove Unwanted Variation (RUV), or upper quartile in the pre-processing step (Mortazavi et al., 2008; Trapnell et al., 2010; Wagner et al., 2012; Risso et al., 2014). FPKM, RPKM, and TPM normalization requires inputting gene length file, which should provide matching gene name and their length in base pair in two-column csv file. RUV normalization requires a list of negative control genes (genes that are stably expressed in all experimental conditions), which should be contained in a one-column csv file. If negative control genes are not available, upper quartile normalization option will replace RUV. The metadata file is required for DE analysis, and should specify experimental conditions (e.g., Control, Treated, etc.) for each genotype listed in the data file. Otherwise, the user can move to the next option to perform/click all available analysis buttons (scatter plot, distribution fit, and Pearson Correlation, etc.) once a data file is loaded (whether normalized or in raw count).

## Data Pre-Processing

Upon submitting data files and all supporting files (gene length, negative control genes, and metadata table), the user can filter the lowly expressed genes by indicating the minimum expression value and the minimum number of samples that are required to exceed the threshold for each gene. If input data contain raw read counts, user can choose one of the normalization options (FPKM, RPKM, TPM, upper quartile, and RUV) listed upon availability of supporting files. FPKM, RPKM, and TPM option perform normalization for sequencing depth and gene length, whereas RUV and upper quartile eliminate unwanted variation between samples. To check for sample variation, Relative Log Expression (RLE) plots (Gandolfo and Speed, 2018) of input and processed data are displayed for comparison.

## Scatter Plot and Distributions

The scatter plot displays all gene expressions between any two columns selected from the datafile. This is intended to show, transcriptome-wide, how each gene expression varies between any two samples. The lower the scatter, the more similar the global responses and vice-versa (Piras et al., 2014). That is, this option allows the user to get an indication of how variable the gene expressions are between any two samples (e.g., between 2 different genotypes or replicates).

After knowing this information, the next process is to make a distribution (cumulative distribution function) plot and compare with the common statistical distributions. As gene expressions are known to follow certain statistical distributions such as power-law or lognormal (Furusawa and Kaneko, 2003; Bengtsson et al., 2005; Beal, 2017; Bui et al., 2018), we included the distribution test function. Previously, we have used power-law distribution to perform low signal-to-noise expression cutoff with FPKM expression threshold of less than 10 (Simeoni et al., 2015). Thus, this mode allows the user to check the deviation of their expression pattern with appropriate statistical distributions to select reliable genes for further analysis.

ABioTrans allows the comparison with (i) log-normal, (ii) Pareto or power-law, (iii) log-logistic (iv) gamma, (v) Weibull, and (vi) Burr distributions. To compare the quality of statistical distribution fit, the Akaike information criterion (AIC) can also be evaluated on this screen.

## Pearson and Spearman Correlations

This mode allows the user to compute linear (Pearson) and monotonic non-linear (Spearman) correlations, (i) in actual values in a table or (ii) as a density gradient plot between the samples.

## Pca and K-Means Clustering

The PCA button plots the variance of all principal components and allows 2-D and 3-D plots of any PC-axis combination. There is also a slide bar selector for testing the number of *k*-means clusters.

## Entropy and Noise

These functions measure the disorder or variability between samples using Shannon entropy and expressions scatter (Shannon, 1948; Bar-Even et al., 2006). Entropy values are obtained through binning approach and the number of bins are determined using Doane’s rule (Doane, 1976; Piras et al., 2014).

To quantify gene expressions scatter, the noise function computes the squared coefficient of variation (Gandolfo and Speed, 2018), defined as the variance (σ^2^) of expression divided by the square mean expression (μ^2^), for all genes between all possible pairs of samples (Piras et al., 2014).

## Differential Expression Analysis

ABioTrans provides users with 3 options to carry out DE analysis on data with replicates: edgeR, DESeq2, and NOISeq (McCarthy et al., 2012; Love et al., 2014; Tarazona et al., 2015). In case there are no replicates available for any of the experimental condition, technical replicates can be simulated by NOISeq. edgeR and DESeq2 requires filtered raw read counts, therefore, it is recommended that the user provide input data file containing raw counts if DE analysis is required using either of the two methods. On the other hand, if only normalized gene expression data is available, NOISeq is recommended.

To better visualize DE analysis result by edgeR and DESeq, volcano plot (plot of log_10_-*p*-value and log_2_-fold change for all genes) distinguishing the significant and insignificant, DE and non-DE genes, is displayed. Plot of dispersion estimation, which correlates to gene variation, is also available in accordance to the selected analysis method.

## Hierarchical Clustering and Heatmap

This function allows clustering of differentially expressed genes. User can either utilize the result from DE analysis, or carry out clustering independently by indicating the minimum fold change between 2 genotypes.

For clustering independently, normalized gene expression (output from pre-processing tab) first undergo scaling defined by $Z_{j}\,\left(p_{i}\right)=\left(x_{j}\,\left(p_{i}\right)-\left({\bar{x}}_{j}\right)\right)/\sigma_{x_{j}}$  where $Z_{j}\,\left(p_{i}\right)$ is the scaled expression of the jth gene, $x_{j}\,\left(p_{i}\right)$ is expression of the jth gene in sample *p_i_*, ${\bar{x}}_{j}$ is the mean expression across all samples and $\sigma_{x_{j}}$ is the standard deviation (Simeoni et al., 2015). Subsequently, Ward hierarchical clustering is applied on the scaled normalized gene expression.

ABioTrans also lists the name of genes for each cluster.

## Gene Ontology

This function is used to define the biological processes or enrichment of differentially regulated genes in a chosen sample or cluster. User can select among 3 gene ontology enrichment test: enrichR, clusterProfiler and GOstats (Falcon and Gentleman, 2007; Yu et al., 2012; Kuleshov et al., 2016).

The user needs to create a new csv file providing the name of genes (for each cluster) in 1 column (foreground genes). Background genes (or reference genes), if available, should be prepared in the same format. Next, the sample species, gene ID type (following NCBI database (Clough and Barrett, 2016)) and one of the three subontology (biological process, molecular function, or cellular component) need selection. The output results in a gene list, graph (clusterProfiler), and pie chart (clusterProfiler and GOstats) for each ontology.

## Typical Analysis Time Estimation

The loading time of ABioTrans for a first time R user is about 30 min on a typical Windows notebook or Macbook. This is due to the installation of the various R-packages that are prerequisite to run ABioTrans. For regular R users, who have installed most packages, the initial loading can take between a few to several minutes depending on whether package updates are required. Once loaded, the subsequent re-load will take only a few seconds.

The typical time taken from pre- to post-processing using all features in ABioTrans is between 10–20 min. Table 1 below highlights the typical time taken for each execution for 3 sample data deposited in ABioTrans Github folder (*zfGenes*, *Biofilm-Yeast*, and *Yeast-biofilm2*).

**TABLE 1:** Time comparison of functionalities for different test data.

**Type of analysis**		**Time (s)**		
		Test 1^*^	**Test 2#**	**Test 3^∧^**
Pre-processing	TPM/RPKM/FPKM and RLE plot	−	−	0.6⁢s
	Upper quartile normalization and RLE plot	−	0.5⁢s	0.6⁢s
	RUV normalization and RLE plot	1.7⁢s	−	−
Scatter plot		0.01⁢s	0.01⁢s	0.01⁢s
Distribution fitting (for all 6 distributions)		4.3⁢s	3.1⁢s	2.5⁢s
Correlation matrix		0.01	0.01⁢s	0.01⁢s
PCA calculation and plotting		0.01	0.01	0.01
DE analysis	edgeR	7.89	1.52⁢s	5.23⁢s
	DESeq2	15.4⁢s	3.1⁢s	11.3⁢s
	NOISeq	29.6⁢s	22.87⁢s	31.0⁢s
Heat map and hierarchical clustering	DE (using edgeR result) (5 clusters)	0.36⁢s	1.7⁢s	0.25⁢s
	Independent (5 clusters)	30.4⁢s	7.7⁢s	4.6⁢s
Noise		3.2⁢s	1.3⁢s	3.9⁢s
Shannon entropy		0.03⁢s	0.02⁢s	0.08⁢s
GO analysis (using edgeR result)	clusterProfiler	20.2⁢s	10.3⁢s	9.1⁢s
	GOstats	26.6⁢s	10.2⁢s	12.3⁢s
	EnrichR	−	−	−

^*^Risso et al., 2014: GEO accession number: GSE53334. #Bendjilali et al., 2017: GEO accession number: GSE85595. ^∧^Cromie et al., 2017: GEO accession number: GSE85843.

ABioTrans has also been compared with other similar freely available RNA-Seq GUI tools, and it

demonstrates better functionalities and capabilities (Supplementary Table S1).

## Summary

ABioTrans is a user-friendly, easy-to-use, point-and-click statistical tool tailored to analyse RNA-Seq data files. It can also be used to analyse any high throughput data as long as they follow the format listed in this technology report. The complete user manual to operate ABioTrans is available as Supplementary Data Sheet S1 in Supplementary Material posted online.

## Availability and implementation

ABioTrans is available at: https://github.com/buithuytien/ABioTrans, Operating system(s): Platform independent (web browser), Programming language: R (RStudio), Other requirements: Bioconductor genome wide annotation databases, R-packages (shiny, LSD, fitdistrplus, actuar, entropy, moments, RUVSeq, edgeR, DESeq2, NOISeq, AnnotationDbi, ComplexHeatmap, circlize, clusterProfiler, reshape2, DT, plotly, shinycssloaders, dplyr, ggplot2). These packages will automatically be installed when the ABioTrans.R is executed in RStudio. No restriction of usage for non-academic.

## Author Contributions

YZ and TTB developed the software tool. KS planned, designed the tool and wrote the manuscript. Supplementary Data Sheet S1 (user manual) was prepared by TTB.

## Conflict of Interest Statement

The authors declare that the research was conducted in the absence of any commercial or financial relationships that could be construed as a potential conflict of interest.

## Supplementary Material

TABLE S1 Comparison of functionalities of ABioTrans with other RNA-Seq tools.
