# Supplementary material for: ABioTrans: A Biostatistical Tool for Transcriptomics Analysis
Source: Front Genet. 2019 May 31;10:499. doi: 10.3389/fgene.2019.00499 (PMC6555198; doi:10.3389/fgene.2019.00499)
Supplement: Supplementary file 2 [file Data_Sheet_1.DOCX]

**Supplementary Information**

**(ABioTrans User Manual)**

**ABioTrans - A Biostatistical tool for Transcriptomics Analysis**

**
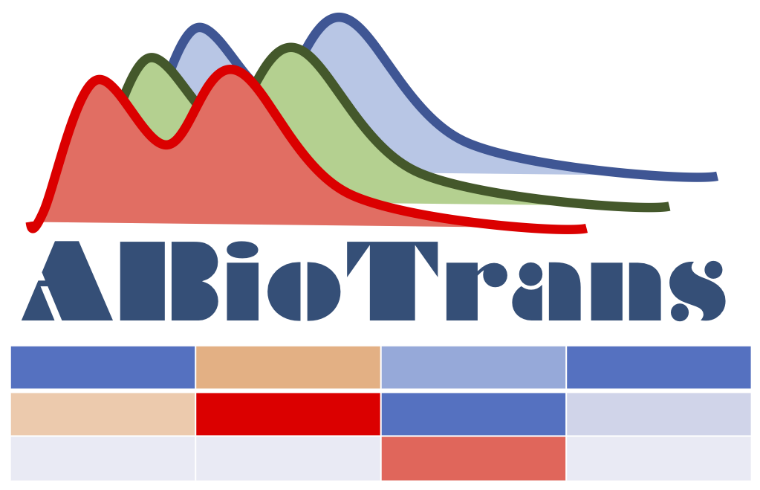
**

Updated 02 May 2019

**by Zou Yutong^1^, Bui Thuy Tien^2^, & Kumar Selvarajoo^2^**

1 Department of Statistics and Applied Probability, National University of Singapore, 6 Science Drive 2, Singapore 117546.

2 Biotransformation Innovation Platform (BioTrans), Agency for Science, Technology & Research (A*STAR), 61 Biopolis Drive, Singapore 138673.

# **Setup**

1. Install R from <https://www.r-project.org/> or Rstudio from <https://www.rstudio.com/>


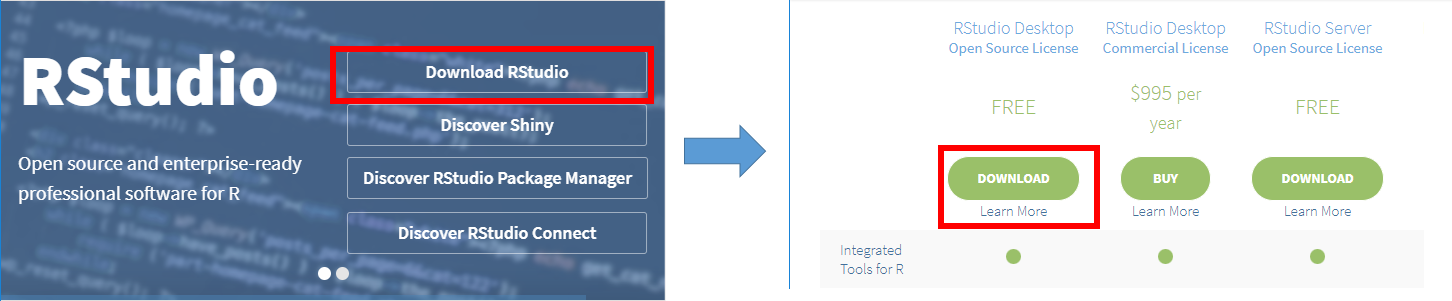


**Figure 1a: Download RStudio**

1. Download ABioTrans-master.zip on GitHub and unzip it. Please do not modify www inside ABioTrans folder.
2. Open the ABioTrans.R file using RStudio and click RunApp button on the top-right corner. Run External opens ABioTrans GUI in your default browser (recommended), whereas Run in Window opens the GUI as a RStudio window. Please note that this user manual works for both Windows and Mac operating system.


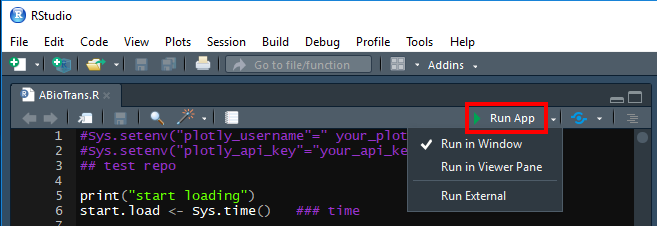


**Figure 1b: Starting ABioTrans from RStudio**

You can start your analysis now!

# **Reading files on ABioTrans**

ABiotrans requires all input files in comma-separated value (.csv) format. The data file in .csv should contain the gene names in rows and genotypes (e.g. wild type, mutants or control, treatments, etc.) in columns, which is similar to the standard transcriptome data file format in GEO database.

Supporting files (if applicable) include gene length, list of negative control genes, and metadata file. A number of normalization options are provided in the pre-processing tab depending on the availability of supporting files: RPKM, FPKM, TPM (requiring gene length), RUV (requiring negative control genes), and Upper Quartile (no supporting file needed). The metadata file is required for differential expression analysis, and should specify experimental conditions (e.g. Control/Treated, time 1/time 2/ time3, etc.) for each genotype listed in the data file. Otherwise, the user can move to the next option to perform analyses (e.g. scatter plot, distribution fitting, Pearson correlation, etc.) once the data file is loaded (whether normalized or in raw count).

| 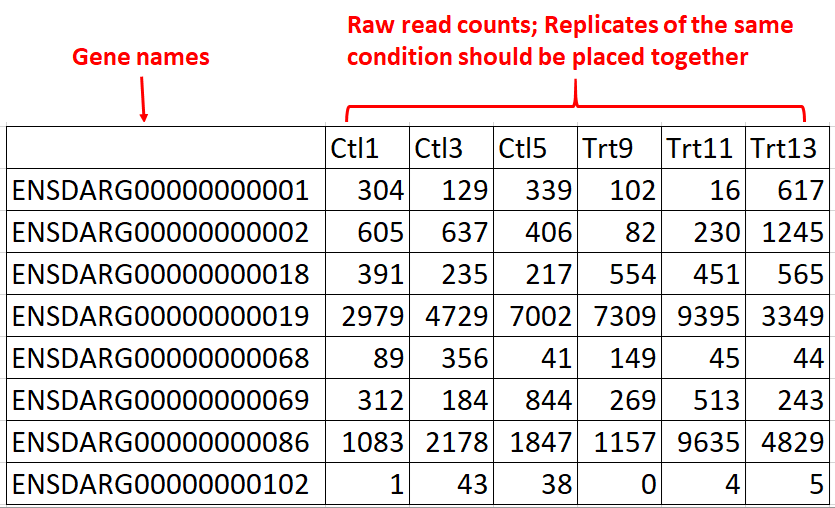  **Figure 2a: Required format for raw counts data file** |
| --- |

| 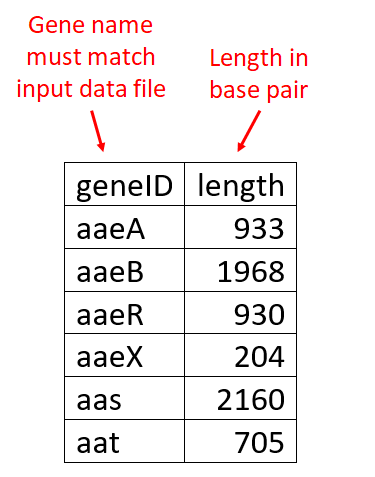  **Figure 2b: Gene length file format** | 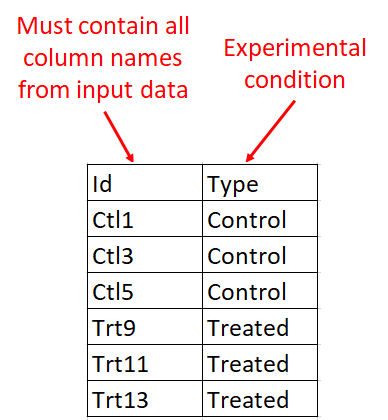  **Figure 2c: Metadata file format** | 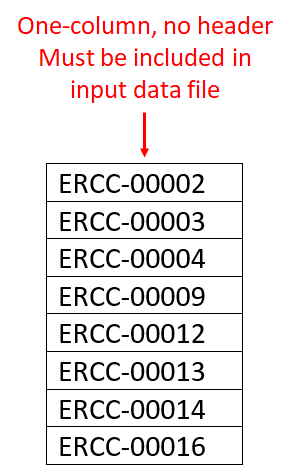  **Figure 2d: Negative control gene file format** |
| --- | --- | --- |

In this tutorial, we will be using the zebra fish data from NCBI GEO database GSE53334: <https://www.ncbi.nlm.nih.gov/geo/query/acc.cgi?acc=GSE53334> . The data has been assembled and converted to csv files in the test data folder <https://github.com/buithuytien/ABioTrans/tree/master/Test%20data> .

Once data files and supporting files are loaded into ABioTrans, user can press Submit button and ABioTrans will automatically proceed to the next tab Preprocessing. In case user accidentally upload wrong data file or supporting files, user can overwrite each of them by uploading new files, or press the Reset button to erase all uploaded files and reset the whole program.


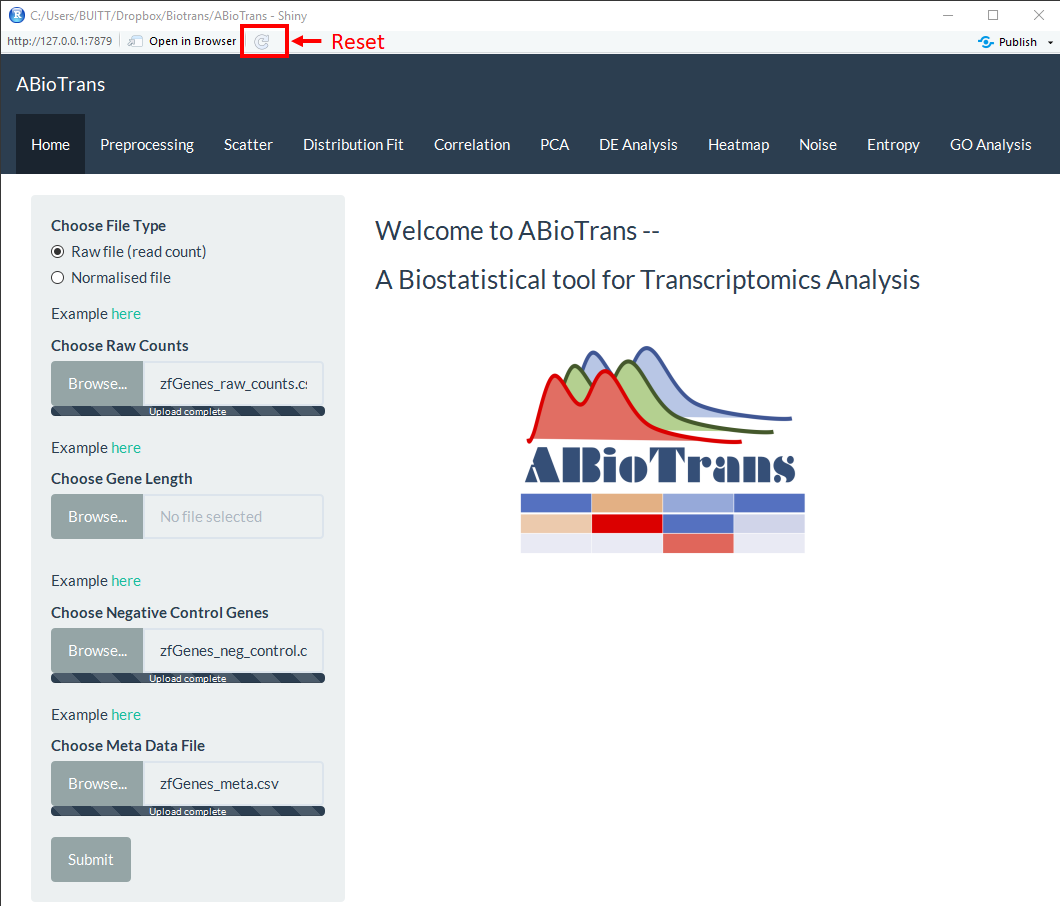


**Figure 2e: Home page and file upload – zebra fish data**

# **Preprocessing**

This involves two steps: removing lowly expressed genes and normalizing the remaining gene expression. First, the user need to specify cut-off expression values (which must be in same unit to the input data file - either raw read counts or normalized expression), and the minimum number of data columns that must exceed the threshold value. Normalization methods are available upon the availability of supporting data files: normalization for sequencing depth, including TPM and RPKM, requires gene length and normalization for sample variation, including RUV, requires negative control genes. Relative Log Expression (RLE) plots of raw and processed data are displayed to visualize the effects of normalization.

In this tutorial, we carried out RUV normalization to remove the unwanted variation between samples (also known as batch effect correction, or between lane normalization). The rest of the analysis pipeline will be implemented on this filtered, RUV-normalized data.


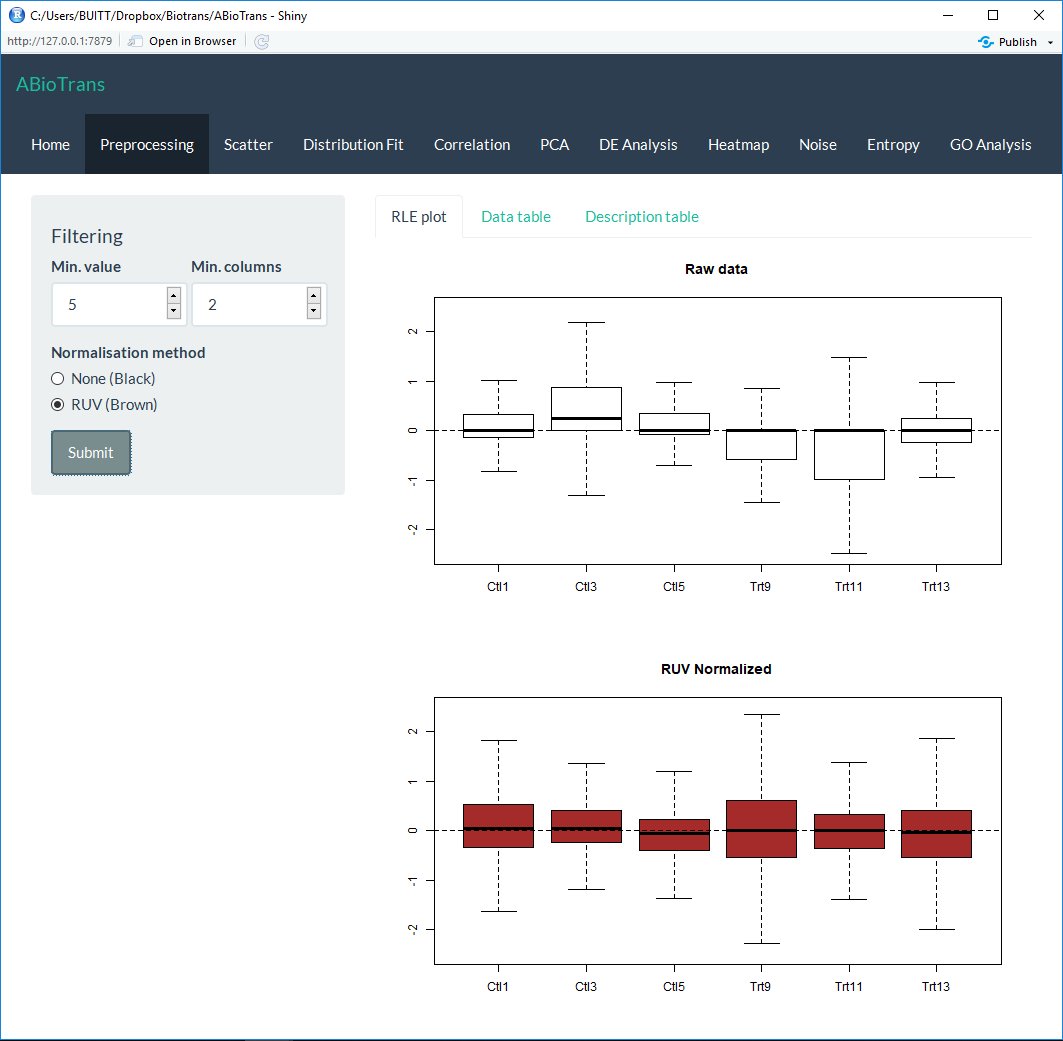


**Figure 3: Preprocessing panel with RLE plots of raw data (upper figure) and filtered, RUV-normalized data (lower figure). Gene expression with minimum five counts in at least two columns were retained**

# **Scatter plot**

Scatter plot compares any 2 samples (or 2 replicates) by displaying the respective expression of all genes in 2D space. Log transformation on the values usually provides better visualization due to the natural skewness of gene expression data. The user can choose to download each single scatter plot, or to download all pairs of samples scatter plot in one PDF file, which may take some time to run.

It is recommended to perform reads normalization (TPM, RPKM, FPKM) for this step (and so does distribution fitting, correlation, hierarchical clustering, noise and entropy). However, since gene lengths are not available for this data set, the tutorial will use RUV- normalized data for all the analysis


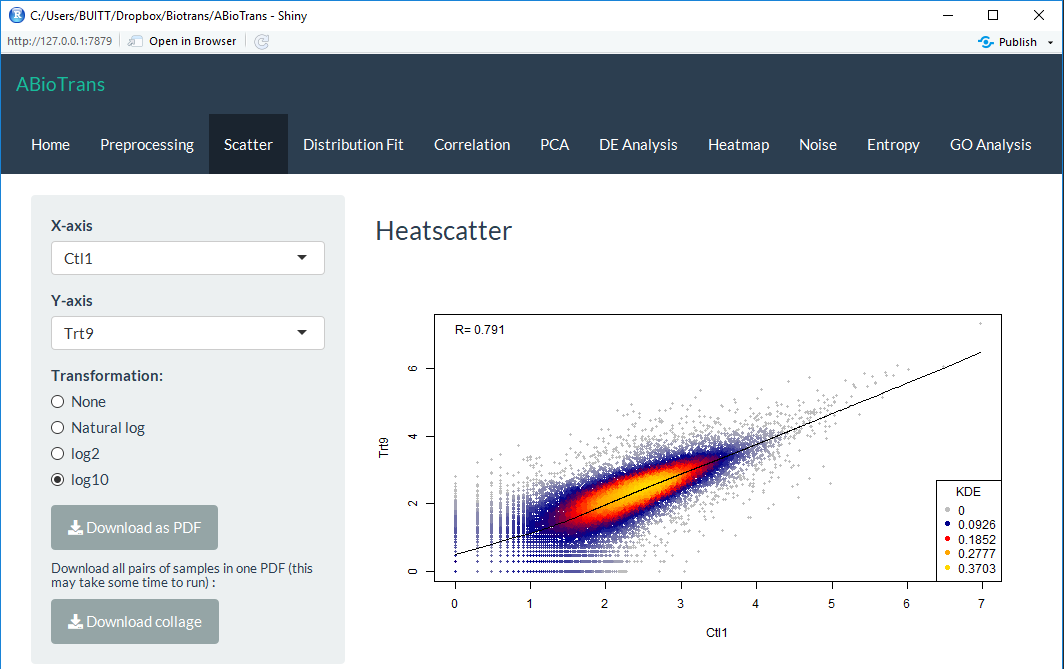


**Figure 4: Scatter plot between Ctrl1 and Trt9 samples with kernel density estimation (KDE)**

# **Distribution fitting**

Distribution fitting compares the gene expression to a number of statistical continuous distributions. To visualize the comparison, ABioTrans displays the Cumulative Distribution Function of the preprocessed gene expression data with the user-selected theoretical distributions. Once it is confirmed that the gene set follow a distribution, it is a good practice to check the quality of the selection by evaluating the Akaike information criterion (AIC). AIC table is also provided in AIC table tab to show the best fitted distribution in each sample


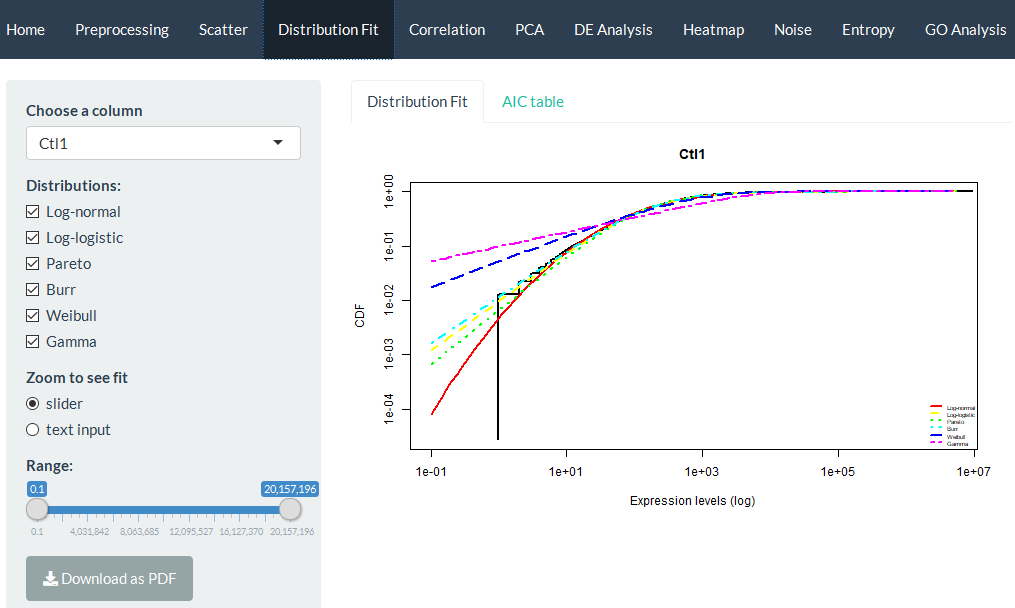


**Figure 5a: Comparing Cumulative Distribution Functions of raw count data (black) with lognormal (red colour), log-logistic (yellow colour), Pareto (green colour), Burr (cyan colour), Weibull (blue colour) and gamma (purple colour) distributions in Ctrl 1 replicate.**


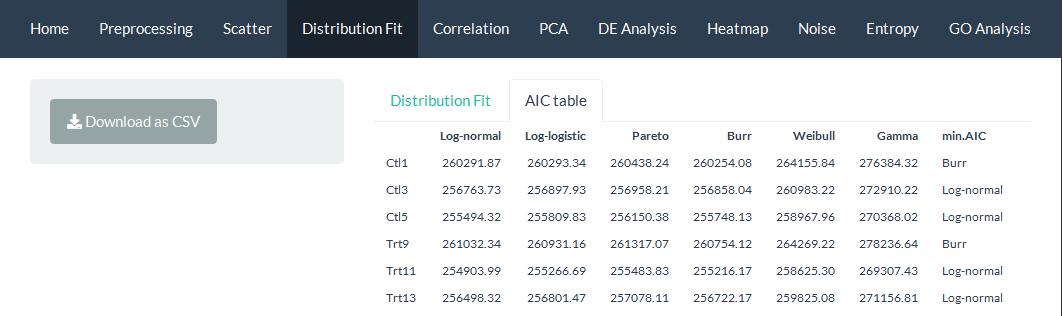


**Figure 5b: Table of AIC values for 6 distributions (Log-normal, Log-logistic, Pareto, Burr, Weibull, and Gamma) in all 6 replicates**

# **Correlation analysis**

In this panel, Pearson and Spearman correlations between any two samples are computed. The correlation values are presented in numeric table (Correlation matrix tab) and heatmap (Correlation heatmap and Correlation plot tab).


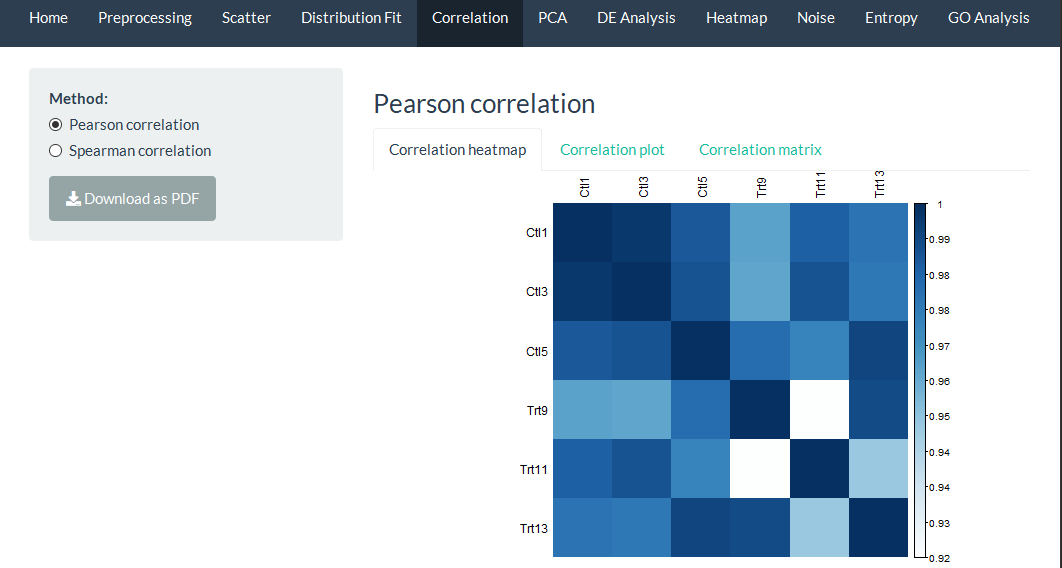


**Figure 6: Heatmap of Pearson correlation between all pairs of replicates**

# **PCA and K-means clustering**

PCA is a linear dimensional reduction method, which is utilized to visualize gene expression data on *N*-dimensional space. By default, expression data from all genes are taken to compute PC values; however, the user can visualize a subset of genes on this PC space by choosing a gene sample size and a gene sample order. For example, to visualize the top 1000 expression genes, the gene sample size to choose is 1000, and the gene sample order is descending. ABioTrans displays the variance percentage of all principal components (scree plot) in PCA variance tab, 2D plot of any PC-axis combination in PCA-2D plot tab and 3D plot in PCA-3D plot tab. K-means clustering is available for PCA 2-D and 3-D plots.


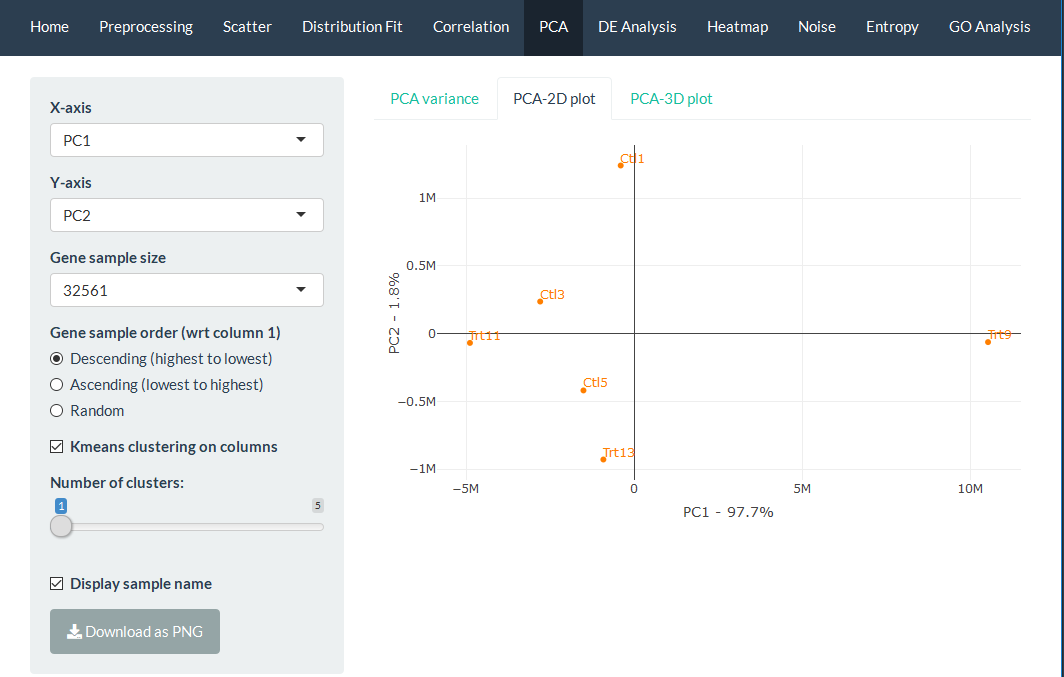


**Figure 7: RUV-normalized count data of 6 replicates on PC1-PC2 space**

# **Differential Expression Analysis**

ABioTrans provides 3 Differential Expression (DE) Analysis methods for multiple replicate dataset: edgeR, DESeq2 and NOISeq. For data with single replicate in all experiment conditions, NOISeq method can simulate technical replicates to carry out DE test. Metadata file is required for DE Analysis. Please make sure metadata contains all column names from input data file and match them with experimental condition

For edgeR and DESeq2, raw read counts data file must be provided. For NOISeq, gene expression should be normalized (by selecting normalization method in Preprocessing tab if raw counts file is read, or by directly providing normalized gene expressions)

To carry out the analysis, first the user needs to specify the preferred DE method, the two conditions to compare (condition 2 is compared against condition 1), fold change cut-off value and False Discovery Rate (FDR or adjusted p-value) threshold, then hit the “Submit” button. By default, DE genes uses FDR = 0.05 and 2-fold change. When the computation finishes, table of DE genes, volcano plot of DE result and dispersion plot of input data are displayed in their respective tabs. Please note that volcano plot and dispersion plot are only available for edgeR and DESeq2 methods.

In this tutorial, we perform DE analysis using edgeR; the result will also be used in Heatmap and hierarchical clustering analysis.


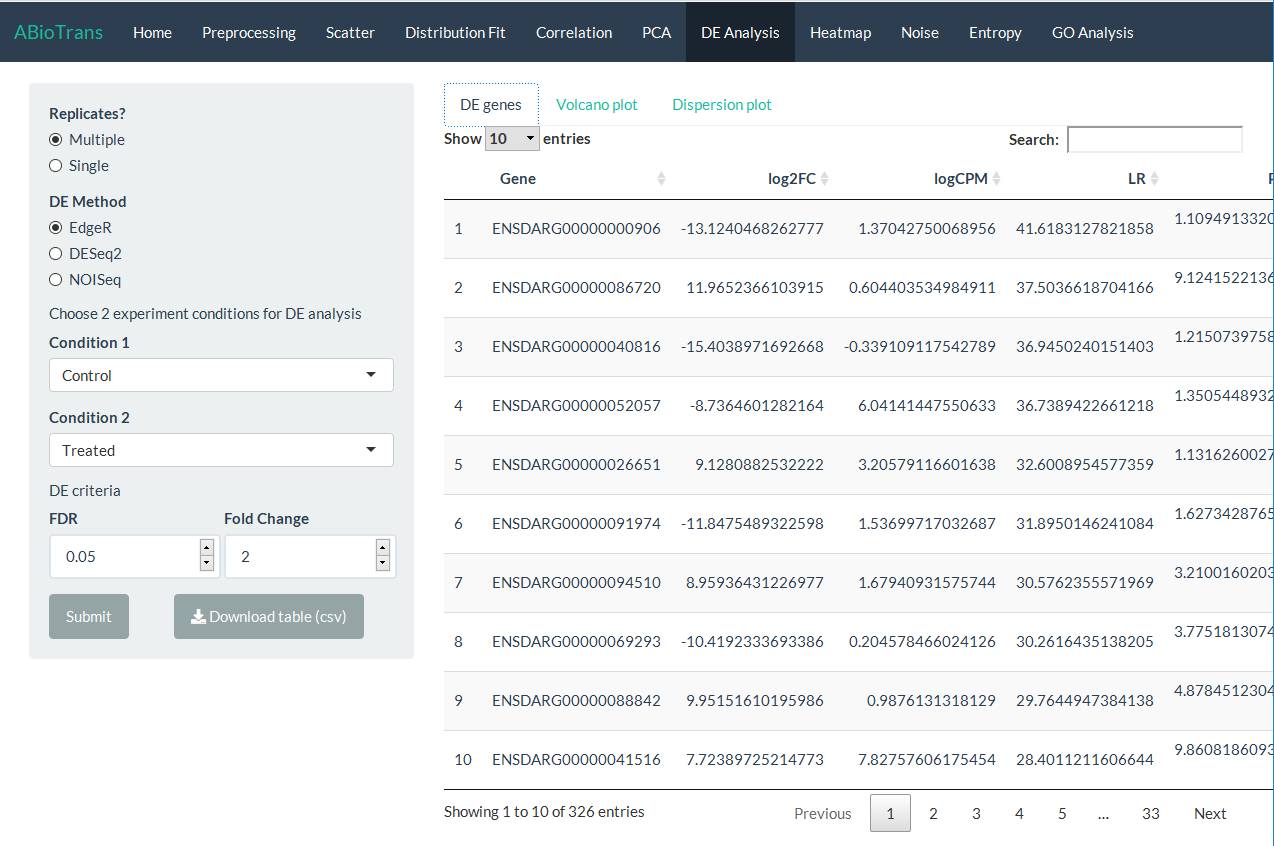


**Figure 8a: Table of DE genes computed by edgeR analysis method between Treated and Control conditions.**


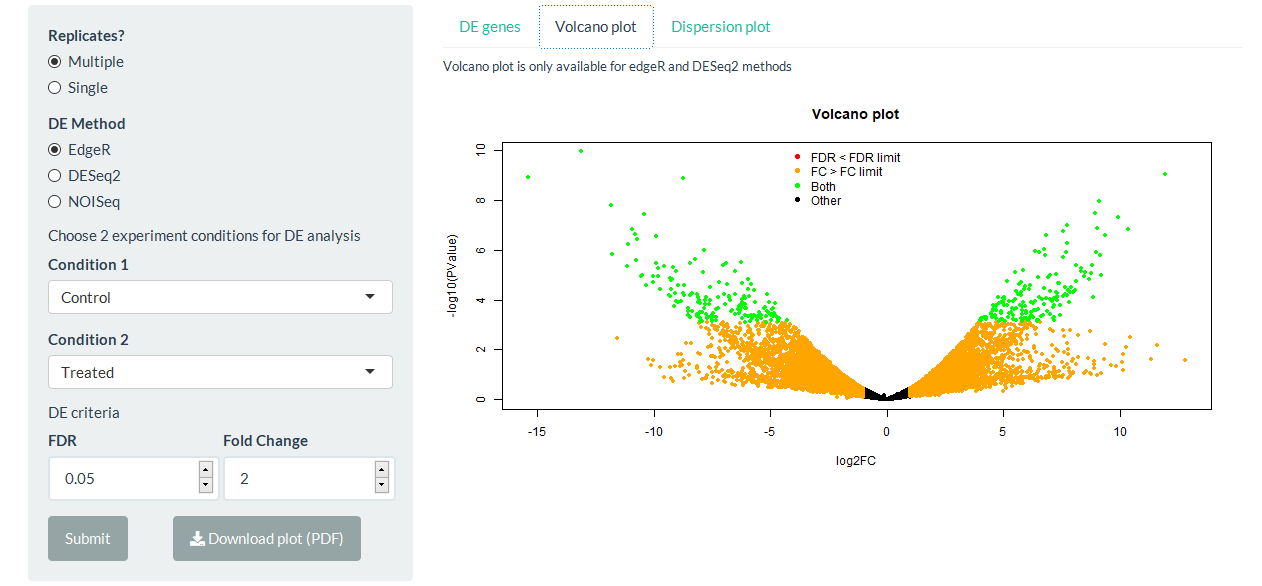


**Figure 8b: Volcano plot from edgeR DE analysis between Treated and Control conditions**


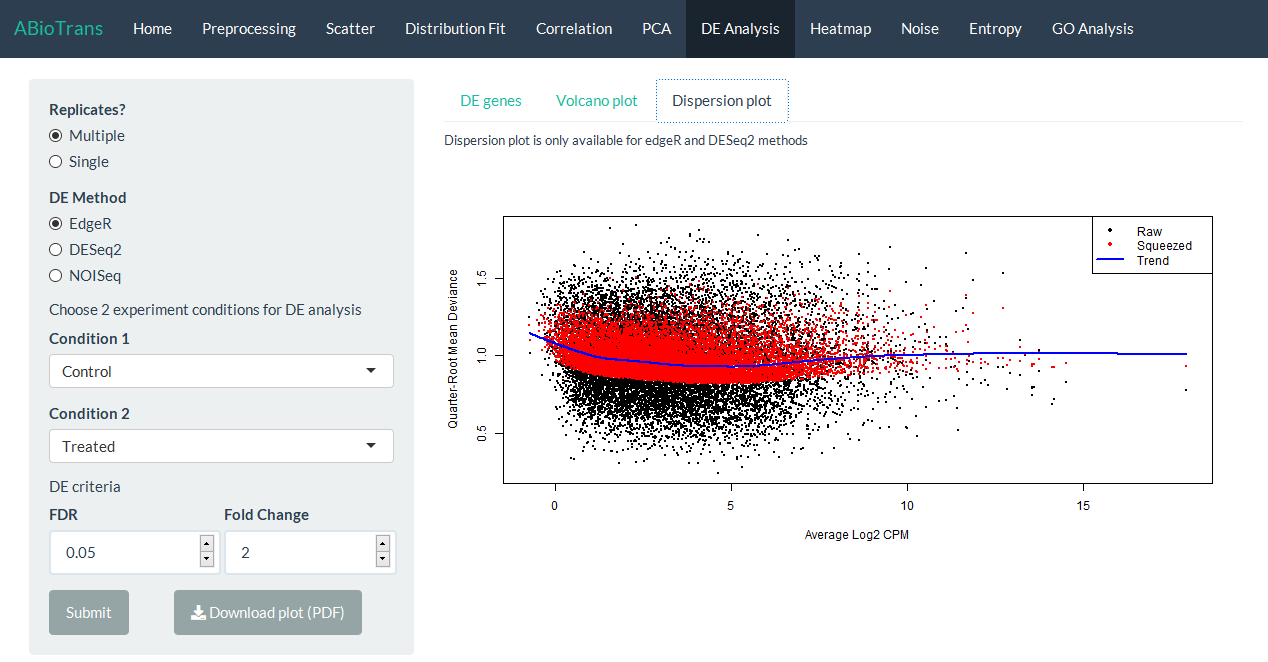


**Figure 8c: Plot of dispersion generated by edgeR method**

# **Heatmap and clustering**

Here, hierarchical clustering on the output of DE analysis using edgeR in the previous section (326 genes) is performed. Alternatively, the user can carry out clustering independently without going through DE analysis by specifying the minimum fold change of gene expression between the 2 samples. ABioTrans also lists the name of genes for each cluster in the Gene clusters tab


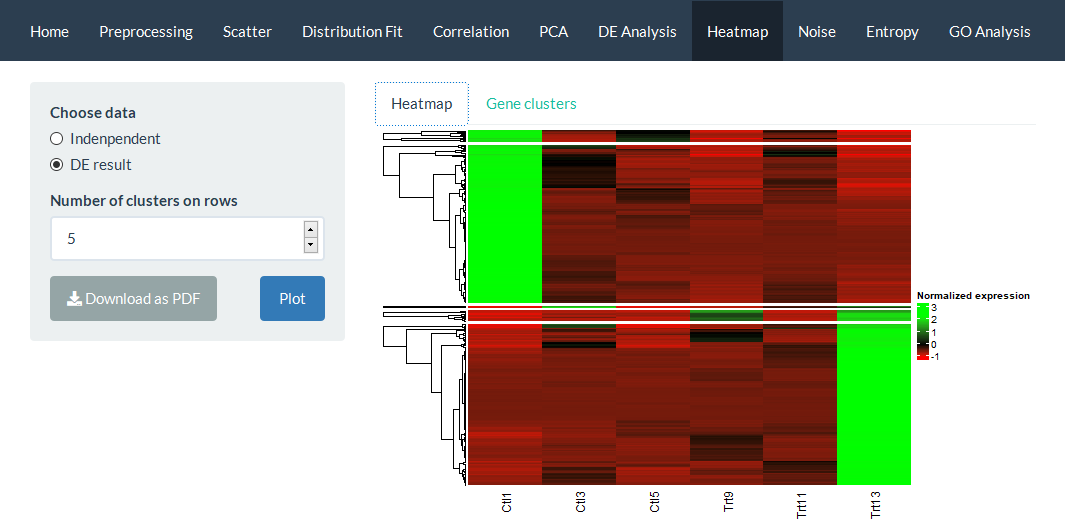


**Figure 9a: Heatmap and hierarchical clustering (5 clusters) on DE genes resulted from edgeR DE analysis**


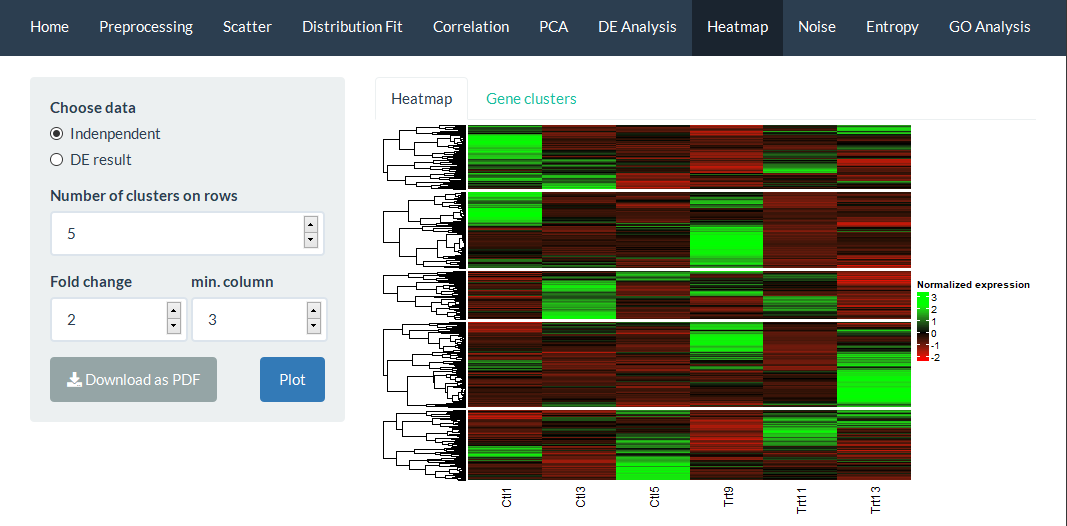


**Figure 9b: Heatmap and hierarchical clustering (5 clusters) on genes with at least 2-fold change in minimum 3 columns (carried out independently from DE analysis)**

# **Noise analysis**

Noise indicates the variability among samples of the same experimental condition. The noise is computed as the squared coefficient of variation, defined as the variance (σ^2^) of expression divided by the square mean expression (μ^2^), for all genes between all possible pairs of samples.

The dataset used in this demo has 3 replicates each for “Control” and “Treated” condition. For replicates option, noise of each condition is displayed. For genotype (average of replicates) option, the noise of Treated condition is computed based on variance of Treated condition against Control condition (which is called noise of Treated condition against Control condition). Similarly, genotypes (no replicate) is to compute the noise of every replicate against one anchor sample.


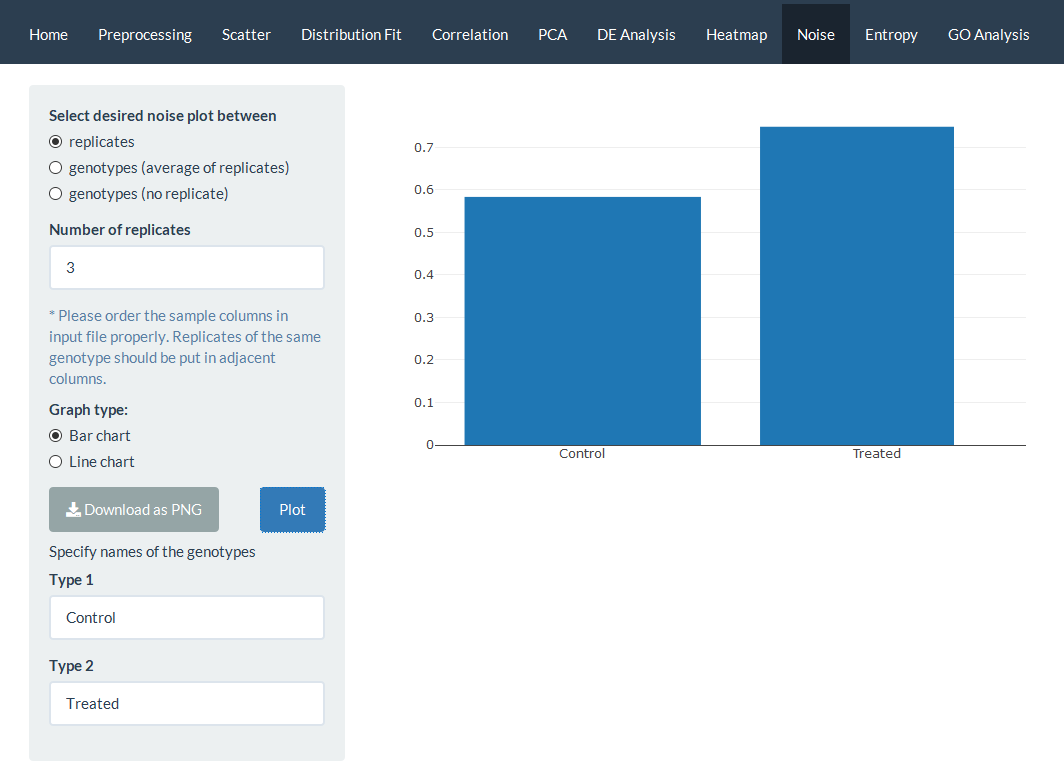


**Figure 10: Noise within Control and Treated groups**

# **Entropy analysis**

Shannon entropy of gene expression in each replicate (X) is defined as

$H\left( X \right)= - \sum_{i-1}^{n} p\left( x_{i} \right)\log_{2}p {(x}_{i})$, where $p\left( x_{i} \right)$ represents the probability of gene expression value $x=x_{i}$. Entropy values were obtained through histogram-based partitioning approach and the number of bins is determined using Doane’s rule: $b\left( X \right)=1+\log_{2} n+\log_{2} (1+\frac{\left| g_{X} \right|}{\sigma_{g}})$, where $g_{X}$ is the skewness of the expression distribution of each sample, and $\sigma_{g}=\sqrt{\frac{6(n-2)}{(n+1)(n+3)}}$.


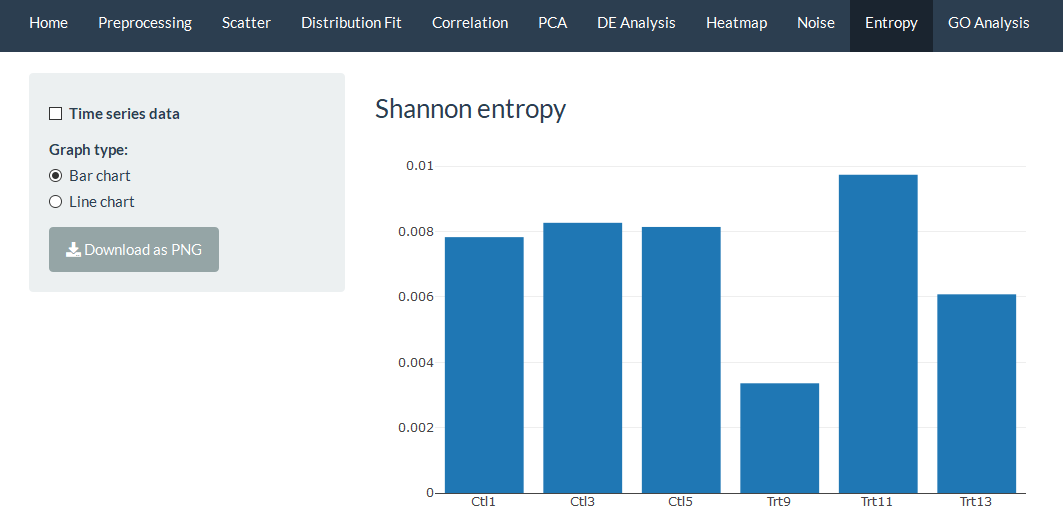


**Figure 12: Entropy of all replicates**

# **Gene ontology analysis**

This analysis results in a list that is significantly enriched in Gene Ontology terms. The user can select among 3 gene ontology enrichment test: clusterProfiler and GOstats, and enrichR:

- Both ClusterProfiler and GOstats uses hypergeometric over-representation test. User need to specify the species, gene identifier and sub-ontology before proceeding to the analysis. clusterProfiler method also implements Benjamini & Hochberg method for p-value correction, and apply a threshold of 0.01 for both p-value, q-value (False Discovery rate) and adjusted p-value. On the other hand, GOstats uses a non-conditional test, with p-value = 0.01 being the only cut-off criteria (hence, GOstats is less stringent than clusterProfiler)
- enrichR requires input gene IDs to be in SYMBOL format. Afterwards user needs to specify the database to perform the analysis.

ABioTrans also displays a pie chart to visualize the relative size of all level-2 ontology terms associated to the gene set in go_pie tab. Note that the pie chart simply displays all GO terms associated to every single gene, which is not the result from the over representation test. Also, there will be overlapping genes in each term displayed (since one gene usually take part in multiple biological functions).

ABioTrans also provides a network visualization to display the mapping between the enriched GO terms and their respective genes in go_graph tab. Please note that the network graph is only available when clusterProfiler method is used.

In this demo, edgeR DE analysis result is saved to .csv file to local drive (file named *zfGenes_DE.csv* from test data folder), and then loaded to GO Analysis tab as list of DE genes. The data used in this demo is in ENSEMBL format, from Danio rerio species (Zebra fish). The demo performs a GOstats test with biological process GO terms


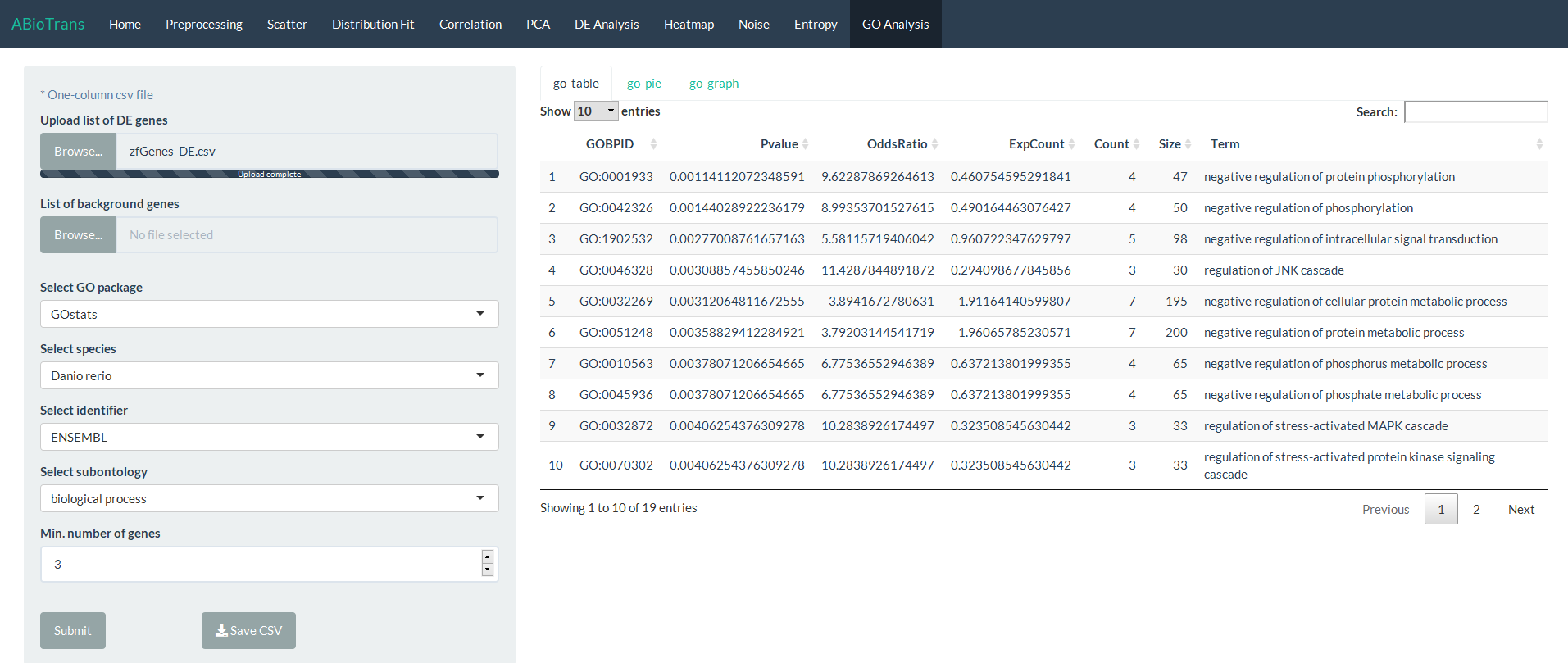


**Figure 13a: Enriched biological processes in DE genes (that resulted from edgeR DE analysis)**


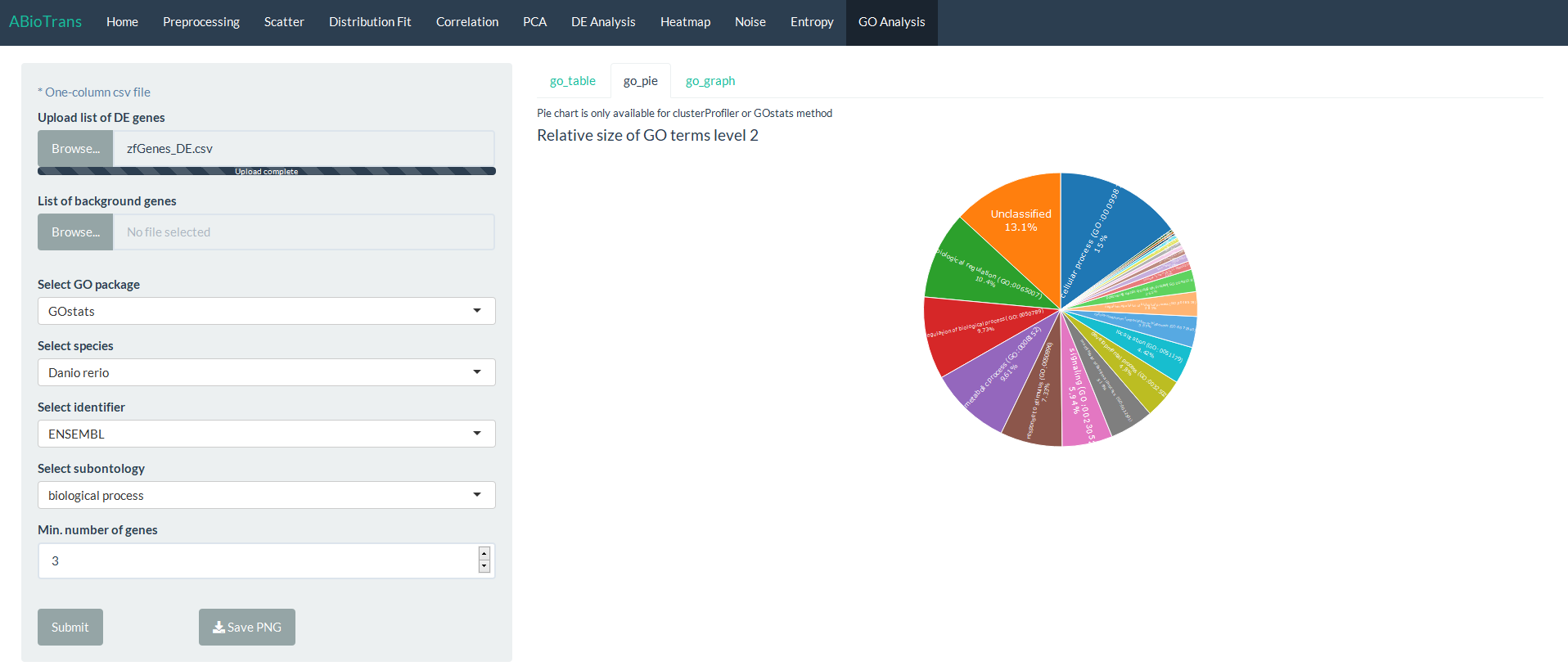


**Figure 13b: Pie chart of all associated biological processes to input DE gene set**
